# Creating a Vision for Education Leadership

**DOI:** 10.5811/westjem.2017.11.35301

**Published:** 2017-12-14

**Authors:** Daniel R. Martin, Felix Ankel, Robin R. Hemphill, Sheryl Heron, Sorabh Khandelwal, Chris Merritt, Mary Westergaard, Sally A. Santen

**Affiliations:** * The Ohio State University, Department of Emergency Medicine, Columbus, Ohio; † Healthpartners Institute, Bloomington, Minnesota; ‡ National Center for Patient Safety, Veterans Health Administration, Washington DC; § Emory School of Medicine, Department of Emergency Medicine, Atlanta, Georgia; ¶ Alpert Medical School of Brown University, Department of Emergency Medicine, Department of Pediatrics, Providence, Rhode Island; || University of Wisconsin, Department of Emergency Medicine, Madison, Wisconsin; # Virginia Commonwealth University School of Medicine, Richmond, Virginia

## BACKGROUND

Academic emergency physicians are driven to become master clinicians while honing their skills in mission areas such as education, research and administration. Many faculty members try to pursue the triple threat of education, service and research; however, excellence in all three areas is difficult to achieve. The first step to excelling in the education domain is to clearly define one’s goals and articulate a strategy to achieve them. To be successful, you must define your vision, mission and core values (VMCV).

As the field of emergency medicine (EM) matures, its education leaders are increasingly recognizing the importance of defining personal and shared visions, core purpose (mission) and core values. In *The Leadership Challenge*, Kouzes and Posner explain “you must clarify your own vision of the future before you can expect to enlist others in a shared vision.”^1^, ^2^ The authors also summarize the benefits of leaders in organizations who are focused on the future, which includes achieving better performance outcomes both individually and as organizations. It comes as no surprise that most academic medical centers, medical schools and some emergency departments have developed shared visions and mission statements and have identified their core values. These statements highlight the core values of the institutions. Leadership experts such as Warren Bennis, Stephen Covey and Peter Senge emphasize the importance of developing your personal vision for life.^3^,^4^,^5^

This brief innovative report will provide tools and examples to articulate a vision statement for education leadership and the steps needed for implementation. The objective of this innovation is for the readers to develop their own vision, mission and core values, and to begin to consider how they will develop their strategy and platform for implementation. While these VMCV may be aligned with your organization’s VMCV, it is important to define your own. Examples of VMCV from education leaders will be presented. This concept is based on a workshop from the Society for Academic Emergency Medicine (SAEM) in 2017 that was developed by key education leaders in the field of EM.

## OBJECTIVES

This education innovation defines each domain of vision, mission, and core values. The reader is then directed through the steps to define their individual domains. Additionally, nine education leaders worked together to clarify their personalized statements.

1. Education leaders will be able to understand the definitions of VMCV and use these tools to create their personal VMCV.
2. Education leaders will adjust their VMCV to align with that of their division, department or organization.
3. Education leaders will use their VMCV to aid in decision-making and developing their strategic plan and future goals.

## CURRICULAR DESIGN

This educational advance leads learners through the process of defining VMCV and then asking participants to determine their own vision, mission, and core values. This is then followed by participants determining their implementation strategy.

### Developing Your Vision

Your personal vision should be the future state you hope to achieve. The vision statement should incorporate the future state and should be a positive, aspirational view of how the future will be better. Collins and Porras defined the vision as consisting of a core ideology and an envisioned future where the core values are the guiding principles.^6^,^7^ They went on to challenge people to create BHAGs, or “Big Hairy Audacious Goals,” emphasizing that vision statements need to be something to strive for about 10 years in the future.

A stepwise approach can be helpful for developing a vision 8,9 starting by contemplating your purpose in the context of a positive future full of possibilities. This theme can be determined by asking yourself to describe your burning passion or what gets you up in the morning, or what do you envision every time you think about the future? Try and align the vision with that of your organization so that one builds on the other. Your vision should go forward several years and be inspirational, bold, exciting and define your burning passion. Transformational leaders are forward thinking, idealistic, possibility-thinkers and dreamers.

Nearly all recommendations for developing one’s vision incorporate consideration and reflection of one’s past, present and future.^1^

Review of one’s past should especially include themes, patterns, experiences, and beliefs that have helped contribute to one’s successes. Past experiences and successes also help define your most important core values. Attending to the present permits one to take inventory of hot topics or areas where futuristic change is clearly needed. Noting the specific details as well as the patterns pointing toward the future are keys to attending to the present.^1^ The future can be considered by asking yourself what you want to accomplish and why? Dreaming or imagining the limitless possibilities in the future is particularly important in times of rapid change.

The final step is using these reflections, considerations, and ideas to articulate succinctly your one-sentence vision statement and then reviewing this often for direction, motivation and inspiration.

Examples of visions include that of Oprah Winfrey, founder of the Oprah Winfrey Network, who articulated her vision this way: “To be a teacher. And to be known for inspiring my students to be more than they thought they could be.”^10^ Amanda Steinberg, founder of DailyWorth.com wrote her vision: “To use my gifts of intelligence, charisma, and serial optimism to cultivate the self-worth and net-worth of women around the world.”^10^

### Developing Your Mission

The mission statement or purpose should be a concise statement that describes how you will get there and your reason for being. This is the path by which you will achieve your vision. The mission statement should describe what you want to be and do in your profession and how you will accomplish your vision. It should answer questions about what you will do, who it is for and how you will do it. The most classic examples of a core purpose can be seen from organizations such as the Walt Disney Company: “To make people happy;” and Merck & Co Inc, “To preserve and improve life.”^2^

### Developing Your Core Values

Core values help to align your vision and mission and should include the 3–5 values that serve as your guiding principles. Collins and Porras describe organizational core values as the “essential and enduring tenets of an organization.”^6^ The core values of Disney are “imagination and wholesomeness.” Kouzes and Posner describe individual core values as the deeply held beliefs – the values, standards, ethics, and ideals – that drive you.”^1^ You will use these core values to guide decisions and actions. They are your personal “bottom line.”^1^

### Developing Your Implementation Strategy

Your strategy is the method by which you will achieve your vision and mission. This is the practical part of the plan where you think about the goals to be achieved and how you will get there. It is focused on the methods that you feel will be important for accomplishing vision and mission. It is your blueprint that will incorporate specific goals for your success. Your platform is the media or milieu in which you function most effectively. For example, for many education leaders, their platform is social media, while for others it is their personal learning network.^11^,^12^

## IMPACT/EFFECTIVENESS

The table displays the VMCV of several education leaders. Each is unique and approaches education from a different perspective. Some of the education leaders focused more at an organizational level, while others were more narrowly focused. Recent evidence has demonstrated a positive association between well-written mission statements and non-profit healthcare sector performance and firm performance.^12^,^13^ The Gallup organization’s research has demonstrated “success-promoting” and “margin-boosting” benefits of focusing on mission.^14^ They believe that mission drives loyalty, fosters customer engagement, improves strategic alignment and brings clarity by guiding decision making.

In a study by Berg he described an intense commitment to “making the world a better place” that was “almost spiritual” in an organization when symbiotic visions and goals could drive employees and organizations.^15^ Similar recommendations regarding the importance of aligned vision, mission and values have surfaced in healthcare as well.^16^ In a publication by pediatric program directors, personal mission statements were recommended to maintain focus and aid in decision-making and strategic planning to empower academicians to make appropriate trade-offs and reach for new opportunities that were well aligned, while eliminating or declining things that were not.^9^

This innovation provides a stepwise approach for readers to define their vision, mission, and core values. Several examples are described. In general, following preparation, a 60- to 90-minute session like that of the SAEM can be used to develop an initial draft of these statements. Evaluations of the SAEM session noted that all participants noted increased ability to describe vision, mission, strategy and platform afterward. A similar session was used by first-year medical students during “Mission Statement Day.”^17^

First, it is important to remember that the process of creating these statements is not necessarily straightforward. Sometimes it is difficult to identify the key features that belong in the VMCV. Although most references describe the importance of vetting these statements to peers, mentors or supervisors,^9^ it can be unsettling to share these intensely personal statements for fear of criticism. It is particularly hard to create a BHAG. The time spent struggling with the VMCV is time well spent. This investment of your time will help you find a direction by which you can influence and lead in your focus area of education.

Second, it is important to remember that the VMCV are not static. While you may choose to stand with an original vision, it is common to have adjustments as the context changes. Therefore, returning to your statements can be helpful especially in times of transition, as well as to reset or reframe your goals. Finally, some leaders choose to keep their VMCV private while others espouse them publicly. Regardless of how open you choose to be with your VMCV, it is most important that your behaviors demonstrate these statements. Moreover, most leaders operate within a social network; therefore, ensuring that the people you work with know your VMCV is key to teamwork and success.

## Figures and Tables

**Table:** Vision, mission, and core values of selected education leaders.

Education leader	Vision, mission, & core values
Felix Ankel, MD VP, Health Professions Education Healthpartners Institute Professor of Emergency Medicine University of Minnesota Medical School	Vision: Health as it could be, affordability as it must be, through relationships built on trust. (adapted from https://www.healthpartners.com/hp/about/) Mission: To improve health and well-being in partnership with patients, learners, and community. Core Values: Excellence, compassion, partnership and integrity
Robin Hemphill, MD, MPH Chief Patient Safety Officer Director of the National Center for Patient Safety Veterans Health Administration	Vision: Zero preventable harms Mission: Safety through high reliability concepts Core Values: Excellence
Sheryl Heron, MD, MPH Vice Chair of Administrative Affairs Emergency Medicine Assistant Dean of Clinical Education & Student Affairs Emory School of Medicine	Vision: Quality care inclusive of all people for all people regardless of their background. Mission: Advancing diversity, equity and inclusion through engagement of key organizational stakeholders Core Values: Professional and personal connections
Daniel Martin, MD, MBA Professor and Vice Chair of Education EM IM Residency Program Director Department of Emergency Medicine The Ohio State University	Vision: To develop, enlighten and empower others to positively impact patients, learners and colleagues through their passion for education, innovation and leadership. Mission: To use a lens of education and innovation to engage and motivate learners to provide the best education and care possible to our patients. Core Values: Culture of integrity and trust, positive approach, connecting with others, use humor whenever possible
Chris Merritt, MD, MPH Pediatric Residency Program Director Assistant Professor of Emergency Medicine & Pediatrics Alpert Medical School of Brown University	Vision: Sustainable child health, excellence in care of ill and injured children anytime, anywhere. A networked community of lifelong learners and advocates. Mission: To empower newcomers to a community of practice, supported by systems of learning, such that they can contribute to the advancement of the common attitudes, interests and goals of our patients and communities. Core Values: Personal relationships, positivity, humor, continuous improvement.
Sorabh Khandelwal, MD Samuel Kiehl III Professor of Emergency Medicine Residency Program Director Department of Emergency Medicine The Ohio State University	Vision: Flourishing Department and Organization Mission: Promoting resident and faculty development into flourishing individuals to improve learning, academic productivity, patient care, and personal and professional relationships. Core Values: Forgiveness, gratitude, be present, hope, faith, optimism
Sally Santen, MD, PhD Senior Associate Dean of Evaluation, Assessment and Scholarship Virginia Commonwealth University School of Medicine	Vision: Improving health through education Mission: Learner centered, Evidence based, outcomes oriented, continuous improvement, scholarship focused Core Values: Serve, learn, team
Mary Westergaard, MD Vice Chair of Education Emergency Medicine Residency Program Director University of Wisconsin	Vision: Inspiring learners to achieve a higher standard of care: for patients, for the practice of medicine, and for themselves. Mission: To guide learners to fulfillment, and training programs to excellence by attending to humanistic principles. Core Values: Modelling the way, promoting and sponsoring, valuing curiosity, challenging injustice
